# 

**Published:** 1904-08-27

**Authors:** 


NOTICE TO CORRESPONDENTS.

All MSS., Letters, Books for Review, and other matters intended for the Editor should be addressed The Editor, "The Hospital," The Hospital Buildings, 28 & 29 Southampton Street, Strand, W.C.

The Editor cannot undertake to return rejected MS., even when accompanied by stamped directed envelope.

**Notice to Advertisers and Subscribers.**

All Advertisements, Orders for Copies of The Hospital, and Business Communications should be addressed to **THE MANAGER (Not to the Editor),** The Hospital, 28 & 29 Southampton Street, Strand, London, W.C.

The Cost of Subscription, post free, is as follows:—Per week, 3½d.; per quarter, 3s. 3d.; per half-year, 6s. 6d.; per annum, 13s. Foreign and Colonial:—Per annum, 19s. 6d., payable, in all cases, in advance.

**NOTICE.—Vol. XXXV. of "The Hospital" (October 1903 to March 1904), in handsome cloth binding, is now on sale at the office of this Journal, price 10s. 6d. Cases for binding the Numbers contained in this Volume 2s. Index to Vol. XXXV., 3½d., post free.**

**The Monthly part for August is now ready. Price 1s., by post, 1s. 3d.**

### MEDICAL APPOINTMENTS.

Thomas Bell, M.D., Medical Officer of Health to the Shepsted Urban District Council. K. J. Courtenay, L.R.C.P.&S.Edin., L.F.P.S.Glasg., Medical Officer of Health for Wareham. J. H. Freer, M.R.C.S., L.R.C.P., Medical Officer of Health to the Rugely Urban District Council. Thomas Harling, L.R.C.P.&S. Edin., L.F.P.S.Glasg., Medical Officer to the Reeth Rural District Council. R. M. Lyon, M.B., C.M., Medical Officer and Vaccinator for the Parish of Cathcart. S. Herbert Perry, M.D.Lond., M.B., Ch.B.Birm., M.R.C.P., Assistant Physician to the General Hospital, Birmingham. A. Douglas Pringle, M.B., Ch.B.Aberd., Senior Assistant Physician to the Government Asylum, Pietermaritzburg, Natal. T. W. Stanton, M.R.C.S., L.R.C.P., Medical Officer for the Osbournby District of the Sleaford Union. E. G. Wales, M.B., M.R.C.S., L.R.C.P., Medical Officer to the Downham Urban District Council.

### "The Hospital" Institutional Library.

"Hospitals and Asylums of the World." 4 Vols., with a Portfolio of Plans, complete £12 12s.

"Burdett's Hospitals and Charities, 1904." 5s. net.

"Burdett's Official Nursing Directory." 3s. net.

"Cottage Hospitals, General, Fever, and Convalescent." 10s. 6d.

"Uniform System of Accounts for Hospitals and Public Institutions." New Edition. 4s. net.

"Hospital Expenditure: The Commissariat." 2s. 6d.

"A Legal Handbook for the Use of Hospital Authorities." 2s. 6d.

"The Cottage Hospital Case Book or Register of Patients." 10s. and 12s. 6d.

All these are published by the SCIENTIFIC PRESS, Ltd., and may be obtained through any bookseller, or direct from the publishers, 28 and 29 Southampton Street, Strand, London, W.C.

**WE** APPEND PARTICULARS OF A FEW OF OUR NURSING MANUALS, BUT DESIRE TO **CENTRE** YOUR SPECIAL ATTENTION ON THE FACT THAT **WE**

THE
**Scientific Press, Ltd.**
Publish and obtain NURSING TEXT-BOOKS of all descriptions.

We shall be pleased to send you a Copy of our Latest Catalogue on application.

KINDLY NOTE OUR ADDRESS:

The HOSPITAL BUILDING,

28 & 29 Southampton Street,

Strand, London, W.C.

**A HANDBOOK FOR NURSES.**

By J. K. WATSON, M.D.

New and Revised Edition. Profusely Illustrated.

5/- net, 5/5 post free.

**NURSING: ITS THEORY AND PRACTICE.**
By PERCY G. LEWIS, M.D.
A Complete Text-Book of Medical, Surgical, and Monthly Nursing.
3/6 post free.

**NURSING: ITS PRINCIPLES AND PRACTICE.**
By ISABEL ADAMS HAMPTON.
New and Enlarged Edition. Illustrated.
7/6 net, 7/10 post free.

**THE NURSES' PRONOUNCING DICTIONARY OF MEDICAL TERMS AND NURSING TREATMENT.**

Compiled by HONNOR MORTEN.

Fifth Edition, Revised and Edited, with phonetic pronunciation, by MARY I. BURDETT.

2/- post free.

**A COMPLETE HANDBOOK OF MIDWIFERY.**

By J. K. WATSON, M.D.

Over 350 pp., profusely illustrated, with 143 Diagrams.

6/- net, 6/4 post free.

**A PRACTICAL HANDBOOK OF MIDWIFERY.**
By FRANCIS W. HAULTAIN, M.D.
New and Revised Edition. Profusely Illustrated.
6/- net, 6/3 post free.

**THE MIDWIVES' POCKET-BOOK.**

By HONNOR MORTEN.

1/- post free.

**HOW TO BECOME A CERTIFIED MIDWIFE.**
By E. L. C. APPEL, M.B., B.S., B.Sc.Lond.
Contains all regulations for the Midwife and for Local Authorities.
1/- net, 1/1 post free.

**NURSING IN DISEASES OF THE THROAT, NOSE, AND EAR.**
By P. MACLEOD YEARSLEY, F.R.C.S. 2/6 post free.

**OPHTHALMIC NURSING.**
By SYDNEY STEPHENSON, M.B.
New and Revised Edition. Illustrated.
3/6 net, 3/10 post free.

**SURGICAL WARD-WORK AND NURSING.**
By ALEXANDER MILES, M.D
Second Edition. Illustrated. 3/6 net, 3/10 post free.

**GYNÆCOLOGICAL NURSING.**
By G. A. HAWKINS-AMBLER, F.R.C.S. 1/- post free.

**PRACTICAL GUIDE TO SURGICAL BANDAGING AND DRESSINGS.**
By WM. JOHNSON SMITH, F.R.C.S.
Profusely Illustrated. 2/- post free.

**ART OF MASSAGE.**
By Mrs. CREIGHTON HALE.
Second Edition. Profusely Illustrated. 6/- post free.

**MEDICAL GYMNASTICS.**
Including the Schott (Nauheim) Movements. Being a Text-Book of Massage and Mechanical Therapeutics generally. Illustrated.
By AXEL V. GRAFSTROM, B.Sc., M.D. 2/6 post free.

OUR NURSING BOOKS ARE OBTAINABLE OF ALL BOOKSELLERS, OR WILL BE SENT DIRECT FROM OUR OFFICE ON RECEIPT OF REMITTANCE.

